# The Calcium-Calpain-ALIX Axis is the Major Link Between Gasdermin-D Membrane Pores and Terminal Membrane Damage in Pyroptosis

**DOI:** 10.21203/rs.3.rs-10100112/v1

**Published:** 2026-06-29

**Authors:** Gergely Imre, Sylvia Otchere, Himesh Parmar, Kushagra Singh, Lauren Tereshisnki, Natalie Thiex, Adam Hoppe

**Affiliations:** South Dakota State University; South Dakota State University; South Dakota State University, Brookings, United States; South Dakota State University; South Dakota State University; South Dakota State University; South Dakota State University

## Abstract

Pyroptosis is an inflammatory form of regulated cell death driven by gasdermin-mediated membrane pore formation. Although gasdermin D (GSDMD) pores are widely regarded as the executioners of pyroptosis, recent studies demonstrate that pore formation is not necessarily lethal because cells can actively repair membrane damage through the Endosomal Sorting Complex Required for Transport (ESCRT) machinery. The molecular mechanism that converts reversible GSDMD pore formation into irreversible membrane rupture and cell death remains unknown.

Here, we identify a calcium-calpain-ALIX signaling axis that mechanistically links GSDMD pore formation to catastrophic membrane damage. Using primary macrophages, THP-1 monocytes, and HCT-116 epithelial cells, we show that depletion of the ESCRT adaptor ALG-2-interacting protein X (ALIX) abolishes membrane repair, promotes GSDMD accumulation, and markedly increases susceptibility to pyroptotic death. We further demonstrate that GSDMD pores trigger calcium influx, which induces proteolytic cleavage of ALIX. Preventing calcium influx or chelating intracellular calcium blocks ALIX cleavage, reduces GSDMD accumulation, and markedly improves cell survival.

Mechanistically, we identify calpains as the calcium-dependent proteases responsible for ALIX cleavage and establish ALIX as a previously unrecognized calpain substrate. Pharmacologic inhibition or genetic depletion of calpains significantly reduces membrane permeabilization and pyroptotic cell death. Mapping of calpain cleavage sites localizes the major cleavage site within the ALIX V-domain. Importantly, calpain-mediated cleavage disrupts ALIX interaction with the ESCRT-III component CHMP4B, thereby preventing ESCRT assembly and membrane repair. In contrast, calcium depletion or calpain knock down restores CHMP4B recruitment and ESCRT activation

Collectively, these findings reveal the first mechanistic pathway linking reversible membrane GSDMD pore formation to irreversible membrane rupture. We propose that GSDMD pore-induced Ca^2+^ influx activates calpains, disables ALIX-dependent ESCRT repair, and drives the transition from repairable membrane injury to terminal pyroptotic lysis. This pathway represents a potential therapeutic target for inflammatory diseases driven by excessive pyroptosis.

## Introduction

Regulated cell death shape tissue homeostasis, immunity, and disease [1–4]. Pyroptosis is a highly inflammatory regulated death pathway characterized by membrane permeabilization and release of intracellular contents [4–7]. Pyroptosis plays a role in the pathogenesis of chronic inflammatory diseases, including inflammatory bowel disease, and arthritis [8–10]. The main executioner components leading to the loss of membrane integrity in pyroptosis are the members of the gasdermin (GSDM) protein family. [11]. GSDMs are generally activated by protease-mediated cleavage into an N-terminal fragment (N-GSDM), which translocates into the cell membrane and oligomerizes resulting in membrane pores. GSDMD is cleaved by proinflammatory caspases, caspase-1, −4, and − 5 subsequently resulting in GSDMD membrane pores [4–6]. Membrane pore formation is followed by irreversible membrane damage and lytic cell death mediated by factors like ninjurin-1 (NINJ-1) membrane oligomers [12].

**Figure 1:**
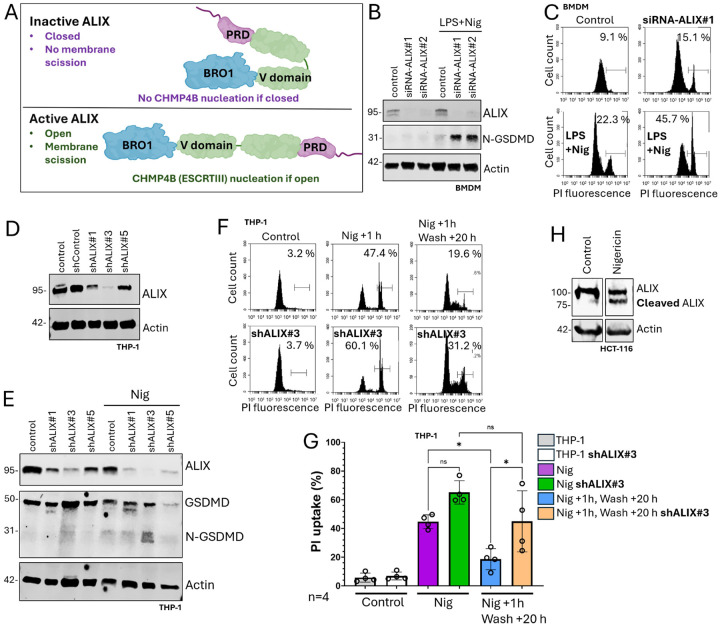
Reduction of ALIX leads to loss of membrane repair and increased cell death susceptibility A) Structure of ALG2 interacting protein X (ALIX) showing Bro1 domain, V domain, and proline rich domain (PRD) domain. Inactive conformation forms a V shape and inhibits Bro1 domain from binding and nucleating charged multivesicular body protein 4B (CHMP4B) and indirectly additional components thus executing membrane scission. Switching to active conformation frees Bro1 domain enabling CHMP4B binding and membrane scission. B) Immunoblot of ALIX and N-GSDMD in control and siRNA-ALIX#1 and #2 knockdown BMDM cells. The cells were primed with 1 μg/ml LPS and treated with 10 μM nigericin (nig) for 1 h. Actin was used as internal loading control. C) Flow cytometry analysis of BMDM control and siRNA-ALIX cells treated as in B. The values show the percentage of cells with increased PI fluorescence intensity (PI uptake). D) Immunoblot of ALIX in control and shRNA-ALIX#1, #3, and #5 stable knockdown THP-1 cells. Actin was used as internal loading control. D) and E) Immunoblot of ALIX and N-GSDMD in control and shRNA-ALIX#1, #3, and #5 knockdown THP-1 cells. In E) The cells were treated with 20 μM nig for 1 h. Actin was used as internal loading control. F) Flow cytometry analysis of THP-1 control and shRNA-ALIX cells treated with nig either for 1 h and detected or washed at 1h and detected at +20 h after wash out. The values show the percentage of cells with increased PI fluorescence intensity (PI uptake). n=4. Significance was tested with one-way ANOVA, p*≤0.05; ns=non-significant H) Immunoblot of full length ALIX and cleaved ALIX in HCT-116 cells treated with nig for 1 h. Actin was used as internal loading control. The values show size in kDa.

Recent studies, including ours demonstrate that GSDM pores can be removed in certain cells [13, 14]. Hence cells possess the intrinsic membrane repair mechanisms, like those involving the Endosomal Sorting Complex Required for Transport (ESCRT) machinery that either remove or repair GSDM pores [13, 14, 15, 16]. The mechanisms of how pore formation leads to either repair or irreversible membrane rupture are only beginning to be unraveled. We previously reported a GSDM pore repair mechanism that is led by the ESCRT protein ALG-2 interacting protein X (ALIX) (alias: PDCD6IP) [14]. ALIX contains 3 functional domains: Bro1, V-domain, and proline-rich domain (PRD) (Fig. 1A) [17]. PRD is required for binding with other ESCRT proteins. ALIX normally exists in an autoinhibitory conformation, in which the PRD domain blocks the Bro-1 domain from nucleating the executioner ESCRT-III. Interaction of PRD with regulatory proteins promotes active conformation [17]. Once ALIX enters the open conformation, ESCRT-III components, including charged multivesicular body protein 4B (CHMP4B) bind to ALIX, the ESCRT-III assembled and executes the membrane scission event leading to vesicle formation [16, 17]. This membrane remodeling process plays a role in exosome shedding and multivesicular body formation [16, 17].

Thus, GSDMD pores are reversible. How GSDMD pores mechanistically trigger irreversible membrane ruptures like NINJ-1 oligomerization [18] or other means of membrane damage remains unclear [12]. Dissecting the molecular cascade linking pore formation to irreversible membrane rupture will provide insights into the switch from sublethal membrane perturbation (by GSDMD pores) to excessive damage and cell death.

Calpains are intracellular Ca^2+−^dependent cysteine proteases that cleave a broad spectrum of substrates including cytoskeletal proteins, membrane proteins, transcription factors, and enzymes (19, 20). While also being implicated in numerous steps during necroptosis and apoptosis (21), whether calpains play a role in the regulation of pyroptotic membrane damage is unknown. Here, we show that calpains are activated downstream of GSDMD pore formation and cleave ALIX, thereby disabling membrane repair and enabling pyroptotic cell death. These findings identify calpain as a novel regulator of pyroptosis, revealing a Ca^2+^ activated calpain–ALIX pathway as a key molecular determinant of pyroptosis susceptibility. Our study takes the first targeted steps towards addressing the mechanistic link between reversible GSDMD pore formation and irreversible membrane damage in pyroptosis.

## Materials and Methods

### Cell culture

HCT-116 (ATCC, Gaithersburg, MD, USA, CCL-247) cells were maintained in Dulbecco’s Modified Eagle Medium (DMEM, Gibco/Thermo Fisher Scientific, Waltham, MA) supplemented with 10% fetal bovine serum (FBS, Biowest, Bladenton, FL, USA, S1480), 100 μg/ml streptomycin and 100 units/ml penicillin (Corning, Glenndale, AZ, USA, 30–002-cl). THP-1 cells (ATCC, TIB-202) were maintained in Roswell Park Memorial Institute (RPMI) −1640 medium (Gibco), supplemented with 10% FBS, 100 μg/ml streptomycin, and 100 units/ml penicillin. The cells were incubated in T-75 cell culture flask at 37°C with 5% CO_2_. For experiments, cells were seeded in 6- or 12-well cell culture plates. For experiments with reduced Ca^2+^ media, Ca^2+−^free DMEM (Gibco) was used and supplemented with 2 mM L-Glutamine, 2% FBS, and antibiotics as described above.

## Bone marrow–derived macrophage (BMDM) isolation and culture

BMDMs were generated from murine femurs as previously described with minor modifications [22]. Briefly, femurs were aseptically isolated, and bone marrow cells were flushed from the marrow cavity using sterile DPBS. Cell suspensions were gently dissociated by pipetting and plated in non-tissue culture–treated Petri dishes containing bone marrow macrophage differentiation medium supplemented with macrophage colony-stimulating factor. Cultures were maintained at 37°C in a humidified incubator with 5% CO_2_. Fresh differentiation medium was added on day 2 of culture. By day 4, adherent macrophages were evident and cultures were expanded as needed. Medium was replaced on day 6 and replenished on day 8 to support continued differentiation and growth.

## BMDM siRNA transfection

BMDMS were cultured in 6- or 12-well plates until 70–80% confluency. The media was replaced with Bone marrow media. 100μl serum-free DMEM was mixed with x-tremeGENE siRNA transfection reagent (Millipore Sigma, #447609301) and 2 μg/ml of ALIX (Pdcd6ip) siRNA’s were mixed and added to the cells as per manufacturer’s instruction. The following ALIX (Pdcd6ip) siRNAs were used: ALIX (Pdcd6ip) siRNA#1: ENSMUSG00000032504 (QIAGEN, Hilden, Germany#EMU015741–20.00UG), ALIX (Pdcd6ip) siRNA#2: ENSMUSG00000170248 (Qiagen, #EHU024581–20.00UG).

## Reagents

Nigericin from *Streptomyces hygroscopicus (S6653)*, *lipopolysaccharide (LPS) (S7850), and PD150606 (S7395) were* obtained from Selleckchem (Houston, Texas, USA) and dissolved in dimethyl sulfoxide (DMSO). BAPTA-AM (B6769) was purchased from Invitrogen (Waltham, MA, USA).

## Plasmid transfection

Cells were cultured in 6- or 12-well plates until 70–80% confluency. The media was replaced with DMEM. 100 μl/ml DMEM was mixed with GeneJuice transfection reagent (ratio: 1 μg DNA: 3 μl GeneJuice0) (Millipore Sigma, Burlington, MA, USA; 70967) and incubated as per manufacturers’ instructions. The following plasmids were employed: pCINeoFlag-ALIX (89859), p3XFlag-CAPN1 (60941), p3XFlag-CAPN2 (60942) (All from Addgene, Watertown, MA, USA).

### Immunoblotting

Cell lysates in Laemmli buffer were run in 7 or 10% SDS-PAGE. The gels were transferred to nitrocellulose membrane. Membranes were probed for indicated primary antibodies overnight and corresponding fluorescent secondary antibodies for 1 h (Licor, Lincoln, Nebraska, USA). The membranes were detected by using Odyssey Fc (Licor). Following primary antibodies were used: Beta-actin (Abcam, Waltham, MA, USA, ab8226) GSDMD cleaved N-terminal (Abcam, ab255983), GSDMD (Novusbio, Centennial, CO, USA, NBP2–33422), ALIX (Novusbio, 90201), GAPDH (Millipore Sigma, SAB4300645), α-spectrin (Cell Signalling Technologies (CST), 2122S), calpain-1 (CST, 2556S), calpain-2 (CST 2539S), Flag (Millipore Sigma, F1804), Caspase-1 (CST 2225T), Caspase-2 (CST, 2224T), Caspase-3 (CST, 9664T), Caspase-4 (CST, 4450S).

## Membrane permeability detection by flow cytometry

0.5 × 10^6^ cells were seeded in a 12-well plate and treated as indicated. Cells were then harvested and incubated with 1 μg/ml propidium iodide (Sigma, P4864) RT for 15 min before subjected to flow cytometry detection with Accuri C6 Plus (BD, Franklin Lakes, NJ, USA), using the FL2 channel (488 nm blue laser/ 585/40 nm band-pass filter). Cell debris (population exhibiting low FSC/FL2 intensity) were excluded from the analysis in FSC/FL2 dot-plot. To measure large-scale membrane damage/rupture, we incubated the cells with FITC-conjugated dextran 500 000 MW (Life Technologies, OR, USA, D7136) (3 μg/mL) for 30 min at 37°C. Cells were washed three times with ice-cold PBS prior to analysis. FITC/FL1 channel (488 nm blue laser/ 533/30 nm band-pass filter) was used.

## In vitro ALIX cleavage assay

Either 0.5 μg human recombinant (hr) full-length ALIX (PDCD6IP-398HFL) or its truncated variants (PDCD6IP-8545H (559–718aa), PDCD6IP-2793H (402–652aa), PDCD6IP-27933TH (1–392aa) (all from Creative Biomart, NY) were mixed with either hr active calpain-1 (Millipore Sigma, Q1A120-DFRZ) or hr active caspases: caspase- 1 (Enzo life Sciences, NY, USA, ALX-201–056), caspase-2 (R&D systems, 702C2010CF), caspase-3, (Enzo life Sciences, ALX-201–059), and caspase-4 (Enzo life Sciences, ALX-201–093A). The ALIX variants and the hr active proteases were mixed in either calpain (Millipore Sigma, Q1A120-DFRZ) or caspase buffer (50 mM HEPES, 10% glycerol, 100 mM NaCl, 10 mM DTT, pH 7.4) respectively to a final volume of 30 μl. Following 3 h incubation at 37°C, the reaction was terminated by the addition of Laemmli buffer. The samples were subjected to immunoblotting to evaluate the cleavage pattern.

### Alamar Blue cell viability assay

0.1 × 10^6^ cells were seeded in 96-well plates and subjected to treatments and incubation conditions as described in the corresponding figure legends. 11 μl of Alamar blue 10 × solution (Fisher Scientific, Waltham MA, A50100) was added to each 100 μl cell suspension / well of a 96 well plate and incubated for 4 h. The fluorescence intensity increase was detected by using a BioTek Synergy2 microplate reader (Thermo Fisher Scientific).

### Flag-ALIX pull down

Cells were collected for each experimental condition, pelleted by centrifugation, and lysed in 500 μl M2 buffer (as per manufacturer’s instruction: Milipore Sigma, M8823). Lysates were subjected to three freeze–thaw cycles using liquid nitrogen, followed by centrifugation at 15,000 × g for 30 min at 4°C to remove insoluble material. For input controls, 50 μl of clarified lysate was mixed with an equal volume of Laemmli sample buffer. Anti-Flag-M2 magnetic beads (Milipore Sigma, M8823) (80 μl per sample) were washed three times with TBS and incubated overnight at 4°C with approximately 450 μl of lysate under continuous rotation. Following incubation, beads were collected on a magnetic stand, and unbound proteins were removed. Beads were washed three times with TBS and resuspended in 50 μl Laemmli sample buffer. Bound proteins were eluted by boiling for 5 min, and the supernatant was collected. Input and pull-down samples were analyzed by immunoblotting.

### Confocal microscopy

5 × 10^5^ cells per well were seeded in a 12-well plate and treated as indicated in the figure legends. After incubation time, the cells were cytocentrifuged onto Colorfrost plus slides (Fisher Scientific, 1255020) using a Cytospin 3 centrifuge (Shandon, Abbey Ward, UK). Slides were fixed in 4% paraformaldehyde (PFA) for 10 min, followed by three washes with PBS. Cells were permeabilized with PBS with 0.1% Triton X-100 for 10 min at RT and subsequently blocked with 10% goat serum for 30 min. Next, slides were washed with PBS 3 × and incubated with primary antibody (1:100 dilution in PBS with 1% BSA) at 4°C in the dark overnight. The following day, cells were washed and incubated for 1 h at room temperature with Alexa 488 conjugated goat anti-rabbit IgG (ABCAM, AB150077) and/or Alexa 647 conjugated goat anti-mouse IgG (CST, 4410S) secondary antibodies, both prepared at 1:100 dilution. Following three PBS washes, cells were mounted with antifade mounting medium with DAPI and covered by coverslips. The following primary antibodies were used: Anti-CD81 (ABCAM, AB59477), Anti-CHMP4B (CST, 42466). Images were acquired using a Leica Stellaris 5 (Leica, Wetzlar, Germany) confocal microscope equipped with 405, 488, 514, 559, and 638 nm laser lines. High-resolution imaging was achieved through pinhole-based optical sectioning using the 63 × oil immersion objective. Laser intensity and gain values were kept constant throughout the samples within the same experiment. The image processing was accomplished by *Leica LAS X Office* (Leica).

#### In silico prediction of calpain cleavage sites

Putative calpain cleavage sites within the ALIX protein sequence were predicted using the *Prosperous Plus* web server. The amino acid sequence of human ALIX (PDCD6IP) (NCBI accession: AAH68454) was submitted to the platform, and cleavage sites were identified using the calpain-1 and − 2 specific prediction algorithm under default parameters. Predicted cleavage sites with high confidence scores (p > 0.9) were selected for further analysis.

### Statistical analysis

Statistical significance was calculated by Student’s t-test (two tailed) or by one-way ANOVA for multiple comparisons using *GraphPad Prism 10* software. All experiments were repeated at least three times. Error bars indicate SD of the mean. Illustrations were created by BioRender.com.

## Results

### Reduction of ALIX leads to loss of membrane repair and increased cell death susceptibility

**Figure 2:**
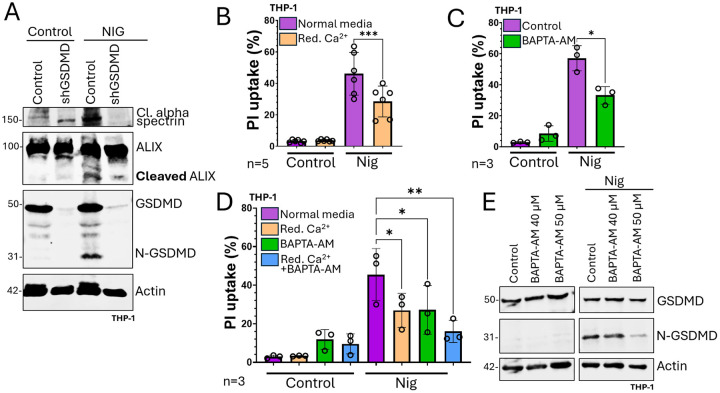
The GSDMD pores induced Ca^2+^ influx leads to ALIX cleavage, increased N-GSDMD level, and pyroptosis A) Immunoblot of cleaved (cl.) spectrin, ALIX, GSDMD and N-GSDMD in control and shRNA-GSDMD stable knockdown THP-1 cells. Actin was used as internal loading contol. B) PI uptake (%) of THP-1 cells in normal vs reduced (red.) Ca^2+^ media was detected by flow cytometry at 1 h after treatment with 20 μM nig. Significance was determined with one-way ANOVA, ***p≤0.005. n=5. C) PI uptake (%) in THP-1 cells was detected by flow cytometry. Cell were pre-treated with BAPTA-AM for 30 min and treated with 20 μM nig for 1 h. Significance was determined with one-way ANOVA, *p≤0.05. n=3. D) PI uptake (%) in THP-1 cells was detected by flow cytometry. Cells were kept in normal or red. Ca2+ media and were pre-treated with 50 μM BAPTA-AM for 30 min, then treated with 20 μM nig for 1 h. Significance was determined with one-way ANOVA, **=p≤0.01; *=p≤0.05. n=3. E). Immunoblot of THP-1 cells harvested 1h or 21 h after treatment with nigericin. Cells were treated with nig for 1 h and then either harvested or washed out and placed back in the incubator for another 20 h. Detection of GSDMD, N-GSDMD and Actin levels. D) Immunoblot of THP-1 cells treated as in C). Detection of GSDMD, N-GSDMD and actin levels. The values show size in kDa.

**Figure 3:**
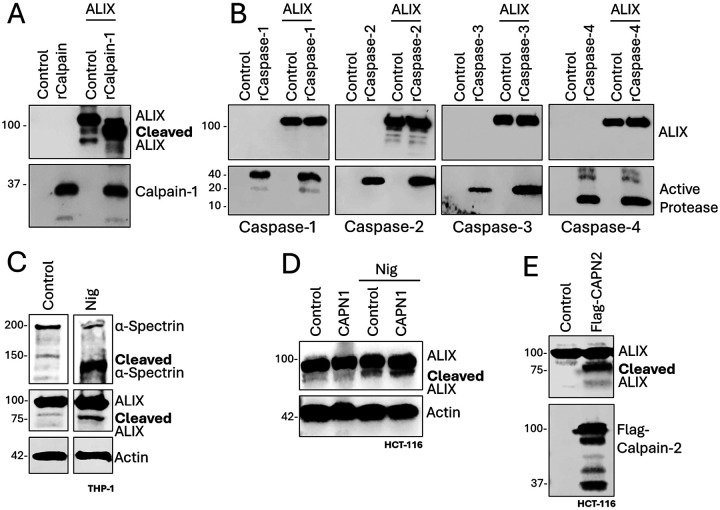
The calcium dependent activation of calpain-1/−2 leads to proteolytic processing of ALIX A) and B) Immunoblot of *in vitro* calpain and caspase cleavage assays of ALIX. C) Immunoblot of THP-1 cells treated with 20 μM nig for 1 h. D) Immunoblot of HCT-116 cells transfected with p3XFlag-CAPN1 for 24 h and treated with nig for 1 h. ALIX and actin was detected. E) Immunoblot of HCT-116 cells transfected with p3XFlag-CAPN2 for 24 h and treated with nig for 1 h. ALIX and calpain-2 was detected. The values show size in kDa.

Previously, we have identified ALIX as an important regulator of GSDMD pore removal in pyroptosis [14]. Based upon this finding, we further investigated pyroptosis in circumstances when ALIX is reduced. To stimulate pyroptosis, we initiated K^+^ efflux, a common trigger of pyroptosis [23, 24]. In bone marrow derived mouse macrophages (BMDMs), we treated cells with LPS and K+ ionophore nigericin (nig). In human monocytic THP-1 and colon epithelial HCT-116 cell lines, we used nig alone to stimulate pyroptosis. The genetic knock down (KD) of ALIX led to significantly increased cell death rate (Fig. 1B–G) and substantially increased N-terminal GSDMD (GSDMD) level in both BMDMs and THP-1 cells (Fig. 1B, E). N-GSDMD is the cleaved fragment of GSDMD leading to GSDMD membrane pores [4, 5]. The repair of membrane pores was entirely halted in cells where ALIX was knocked down (KD) (Fig. 1G and **Suppl. Figure 1**). Hence, this data indicates that N-GSDMD is not removed over time if ALIX is depleted. High ALIX has been shown to correlate with enhanced survival rate in cells, facilitate N-GSDMD removal [14], control tumor progression [25], and result in better prognosis in ulcerative colitis patients [26], hence we set out to investigate how ALIX level is regulated at the cellular level. We observed the occurrence of a smaller size fragment of ALIX in pyroptotic samples appearing around 70–90 kDa (referred to as ‘*cleaved’*) (Fig. 1H, 2A, 3C).

### The GSDMD pores induced Ca2 + influx leads to ALIX cleavage, increased N-GSDMD level, and pyroptosis

GSDMD pores lead to Ca^2+^ influx [27]. To determine the mechanism that results in ALIX cleavage, potential ALIX inactivation, and loss of membrane repair, we investigated the role of GSDMD pores triggered Ca^2+^ influx. Intrigungly, GSDMD stable KD cells did not exhibit cleaved ALIX as compared to wild type cells (Fig. 2A) and cells cultured in reduced Ca^2+^ media (Fig. 2B) or pretreated with a Ca^2+^ chelator BAPTA-AM (Fig. 2C) showed enhanced survival rate. Using the combination of reduced Ca^2+^ and intracellular chelation of Ca^2+^ resulted in a total inhibition of cell death (Fig. 2D). Finally, Ca^2+^-chelation substantially reduced the level of N-GSDMD (Fig. 2E) suggesting an accelerated pore removal mechanism in Ca^2+^-depleted cells.

### The calcium-dependent enzymes, calpains proteolytically cleave ALIX

To determine if the cleaved ALIX fragment is generated by proteases, we tested the cytosolic proteases that can potentially play a role in this process, including caspases and calpains. The *in vitro* protease assays confirmed that the cleavage was accomplished by calpain-1 (Fig. 3A, B, and **Suppl. Figure 2**). Additionally, the calpain-specific substrate α-spectrin [28] was cleaved upon nig stimulation and was blocked in GSDMD KD cells (Fig. 2A), Calpain-1 and − 2 show highly overlapping cleavage profile and specificity, yet they differ in the amount of Ca^2+^ required for activation (μ-molar vs. m-molar respectively) [29]. To test if calpain-1 and − 2 have different affinity towards ALIX, we overexpressed these proteases in cells. Overexpression led to increased ALIX cleavage (Fig. 3D and E). These data together confirm ALIX as a novel substrate of calpain-1 and − 2.

### Calpain knock down reduces pyroptotic membrane damage

**Figure 4:**
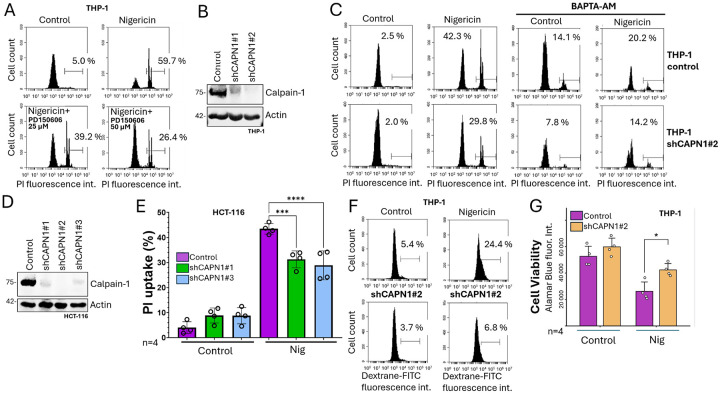
Calpain knock down reduces pyroptotic membrane damage A) The histograms show PI uptake (%) of THP-1 cells detected with flow cytometry. The cells were pre-treated with PD150606 for 30 min and treated with 20 μM nig for 1h. B) Immunoblot of THP-1 and THP-1 shRNA-Calpain-1 (shCAPN1#1 and #2. Calpain-1 and actin were detected. C) The histograms show PI uptake (%) of THP-1 and THP-1 shRNA-Calpain-1 (shCAPN1#2) cells detected with flow cytometry. The cells were pre-treated with 50 μM BAPTA-AM for 30 min and treated with 20 μM nig for 1h. Significance was determined with one-way ANOVA, ***p≤0.005. n=5. C) PI uptake (%) in THP-1 cells was detected by flow cytometry. Cells were pre-treated with BAPTA-AM for 30 min0 and treated with 20 μM nig for 1 h. D) Immunoblot of HCT-116 and HCT-116 shRNA-Calpain-1 cells (shCAPN1#1 and #2). Calpain-1 and actin were detected. The values show size in kDa. E) PI uptake (%) of HCT-116 cells detected with flow cytometry. The cells were treated with 20 μM nig for 24 h. Significance was determined with one-way ANOVA, ***p≤0.001, ****p≤0.0001. n=4. F) Dextran-FITC uptake detected by flow cytometry in THP-1 and THP-1-shRNA-CAPN-1 cells in reduced Ca^2+^ media. The cells were treated with 20 μM nig for 24 h. The values show the percentage of cells with large-scale membrane rupture. G) Alamar blue assay shows THP-1 and THP-1 shRNA-Capn1 cells at 5 h after nig treatment. The values indicate relative fluorescence intensity units proportional with the increased viability of the cells. To determine significance Student’s t test was used. *p≤0.05, n=4.

To further explore the role of calpains in the regulation of pyroptosis, we employed a calpain inhibitor PD150606 and generated stable calpain-1 KD cells. PD150606 markedly inhibited membrane permeability (Fig. 4A) in THP-1 cells, and stable calpain-1 KD THP-1 and HCT-116 cells exhibited significant membrane permeability reduction (Fig. 4B–E). Near total inhibition was achieved by using BAPTA-AM in calpain-1 KD cells (Fig. 4C). Large-scale membrane rupture detected by dextran uptake (Fig. 4F) was entirely blocked at 24 h and cell viability was preserved in calpain-1 KD cells (Fig. 4G) supporting the premise that calpain-driven cleavage is the decisive factor towards irreversible membrane rupture and pyroptosis.

### Calpain cleavage of ALIX disrupts its ability to nucleate CHIMP4B and engage in membrane repair

**Figure 5:**
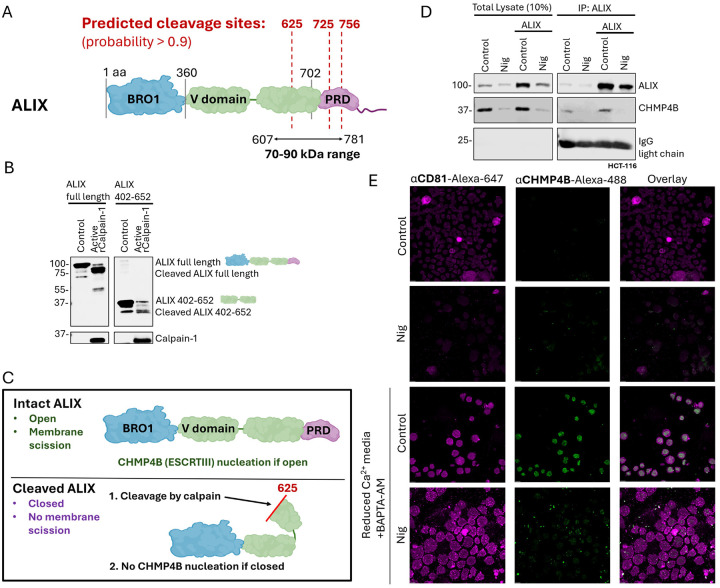
Calpain cleavage of ALIX disrupts its ability to nucleate CHIMP4B and engage in membrane scission and repair A) Structure of ALG2 interacting protein X (ALIX) with the *in silico* predicted calpain cleavage sites (red dashed lines). B) Immunoblot of *in vitro* calpain cleavage assay of full length and truncated ALIX (aa402–652). ALIX and calpain-1 were detected. C) The cartoon shows full length ALIX in open conformation and cleaved ALIX in closed conformation leading to inactivation. D) Immunoblot of flag-ALIX co-immunoprecipitation of HCT-116 cells transfected with pCINeoFlag-ALIX treated with 20 μM nigericin (nig) E) Confocal microscopy images show THP-1 cells in normal and reduced Ca^2+^ media pre-tretaed with BAPTA-AM for 30 min and treated with 20 μM nig for 1 h. CD81-Alexa-647 (magenta) and CHMP4B-Alexa-488 (green) are shown. White demonstrates colocalization of CD81 (cell membrane) and CHMP4B. A single z-plane is presented. Scale bar=10 μM.

**Figure 6:**
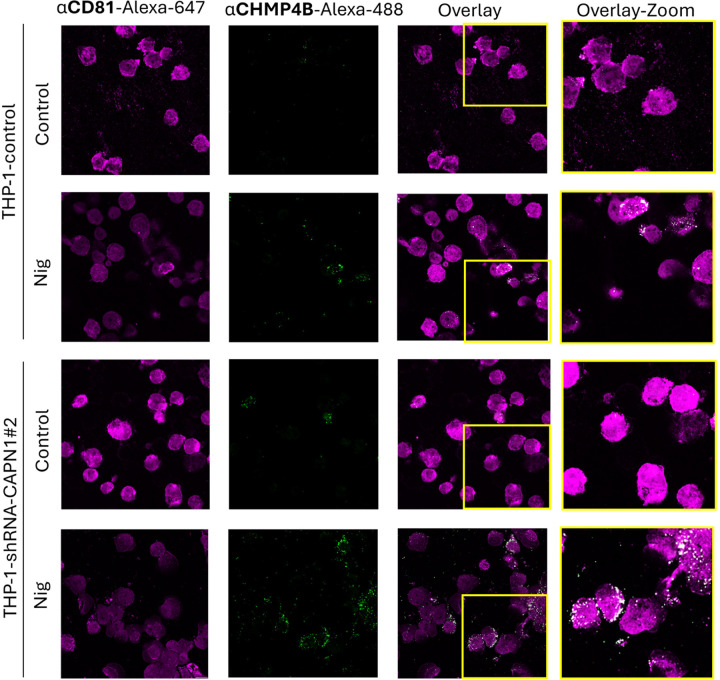
Calpain knock down increases CHMP4B nucleation A) Confocal microscopy images show THP-1 and THP-1 shRNA-Calpain 1 (CAPN1) cells treated with 20 μM nig for 1 h. CD81-Alexa-647 (magenta) and CHMP4B-Alexa-488 (green) are shown. White demonstrates colocalization of CD81 (cell membrane) and CHMP4B. A single z-plane is presented. Scale bar=10 μM.

**Figure 7:**
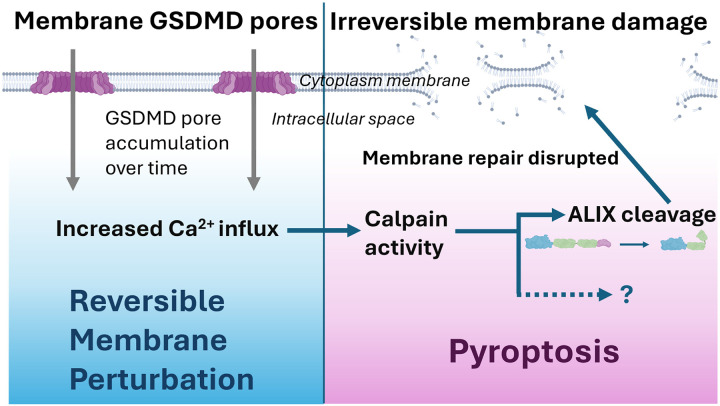
The mechanistic link between GSDMD pores and membrane rupture The cartoon shows that Ca2+ influx and calpain activity driven ALIX cleavage represents the mechanistic link between GSDMD membrane pores and irreversible membrane rupture.

Using an *in silico* calpain cleavage site prediction tool (prosperousplus.unimelb-biotools.cloud.edu.au), we identified three putative calpain cleavage sites in ALIX with high probability (p > 0.9) within 70–90 kDa range: 625 (ESL▼KKQEG), 725 (PSAPS▼IPT), and 756 (PPTK▼PQPP) (Fig. 5A). To determine which predicted cleavage occurs upon calpain activation, we tested the full-length ALIX and a truncated ALIX variant (402–652) using an *in vitro* calpain assay. The *in vitro* calpain assay showed cleavage in samples containing both the full length and the ALIX 402–652 variants (Fig. 5B) excluding the 725 and 756 sites and confirming the 625 site as the most likely calpain cleavage site in ALIX. ALIX contains 3 functional domains: Bro1, V domain, and proline-rich domain (PRD) [30]. ALIX normally exists in an autoinhibitory conformation, in which the closed (V shape) conformation of the V-domain blocks the Bro-1 domain from nucleating ESCRT-III and executing the membrane scission event [31]. Thus, we propose that calpain-1/−2 mediated cleavage of ALIX disrupts the ability to enter the active conformation and nucleate the ESCRT-III component CHMP4B (Fig. 5C). ALIX co-immunoprecipitation in HCT-116 cells confirmed ALIX-CHMP4B interaction. In line with our previous findings, this interaction was disrupted in nigericin treated cells (Fig. 5D). In addition, as a sign of ESCRT-III nucleation, marked increase in the formation of CHMP4B puncta was observed by fluorescence laser scanning microscopy in Ca^2+^ depleted cells (Fig. 5E) and calpain-1 KD cells (Fig. 6). This data together establishes the role of Ca^2+^ -dependent calpain cleavage as the regulator of ALIX’s function in pyroptotic membrane repair and the link between reversible GSDMD pores and irreversible membrane damage (Fig. 7).

## Discussion

Although GSDMD pore formation is the defining event of pyroptosis [4, 5], the mechanisms governing the transition from reversible membrane pores to terminal membrane rupture remain poorly understood. Here, we identify a calcium–calpain–ALIX signaling axis that links GSDMD pore formation to catastrophic membrane failure.

Previous studies have shown that ESCRT-mediated membrane repair can counter GSDMD-induced damage [13, 14] and delay cell lysis. However, why this repair response ultimately fails has remained unclear. Our data identify calcium-dependent proteolytic inactivation of ALIX as a key mechanism underlying this process. We demonstrate that GSDMD pores induce Ca^2+^ influx, leading to ALIX cleavage, whereas reduction of Ca^2+^ reduces cleaved N-GSDMD accumulation, and improves cell survival. These findings suggest that Ca^2+^ serves a dual role during pyroptosis: while transient Ca^2+^ signaling is required for membrane repair, sustained Ca^2+^ influx promotes irreversible membrane damage.

Although our results identify ALIX as a major calpain substrate during pyroptosis, additional calpain targets may also contribute to membrane destabilization. Calpains cleave numerous cytoskeletal and membrane-associated proteins [19–21], and future studies will be required to define their contribution to pyroptotic membrane damage.

Ca^2+^ represents a dual role in regulation of membrane dynamics. Several membrane remodeling events require elevated Ca^2+^ level [32]. While in general, these pathways require lower Ca^2+^ level than those of needed for calpain activation [33], the time kinetics of Ca^2+^ fluctuations within subcellular level warrant a future line of investigations.

Our genetic studies focused on calpain-1 because suitable reagents for stable calpain-2 knockdown were unavailable. However, the stronger protection observed with combined calpain-1 depletion and Ca^2+^ chelation suggests that calpain-2 may also contribute to this pathway. Although calpain-1 and calpain-2 have largely overlapping substrate specificities, they differ in Ca^2+^ requirements, with calpain-1 activated at low micromolar Ca^2+^ and calpain-2 requiring substantially higher Ca^2+^ levels [29]. The sustained Ca^2+^ influx induced by GSDMD pores may therefore promote activation of both isoforms during pyroptosis.

Finally, GSDMD pores induce both increased Ca^2+^ influx and K^+^ efflux [27]. While our findings establish a dominant role for calcium-dependent calpain activation, we cannot entirely exclude at least indirect contributions from K^+^ loss. K^+^ efflux is a well-established trigger of NLRP3 inflammasome activation [34] and has also been linked to activation of caspase-2 [35, 36] and other stress-response pathways. These mechanisms may cooperate with calcium-dependent signaling to amplify membrane damage and promote pyroptotic progression with positive feedback loop for enhanced NLRP-3 activation.

In summary, our study identifies a previously unrecognized calcium–calpain-ALIX pathway that governs the transition from repairable GSDMD pore formation to irreversible pyroptotic membrane rupture. By demonstrating that calpain-mediated ALIX cleavage disables ESCRT-dependent membrane repair, our findings provide a mechanistic explanation for the collapse of membrane integrity during pyroptosis and highlight this pathway as a potential therapeutic target in inflammatory diseases driven by excessive pyroptotic cell death.

## Supplementary Material

Supplementary Files

This is a list of supplementary files associated with this preprint. Click to download.

- OriginalWesternBlots.pdf
- Slide2.jpg
- Slide1.jpg
